# Transition of fluctuations from Gaussian state to turbulent state

**DOI:** 10.1098/rsta.2021.0097

**Published:** 2022-03-07

**Authors:** Toshiyuki Gotoh, Jingyuan Yang

**Affiliations:** ^1^ Department of Physical Science and Engineering, Nagoya Institute of Technology, Gokiso, Nagoya 466-8555, Japan; ^2^ Research and Education Center for Natural Sciences, Keio University, Hiyoshi, Yokohama 223-8521, Japan; ^3^ Institute of Industry Technology, Guangzhou and Chinese Academy of Sciences, 1121 Haibin Road, Nansha Dis., Guangzhou City, People’s Republic of China

**Keywords:** transition, fluctuations, Gaussian, turbulence, moment, probability density function

## Abstract

Variation of the statistical properties of an incompressible velocity, passive vector and passive scalar in isotropic turbulence was studied using direct numerical simulation. The structure functions of the gradients, and the moments of the dissipation rates, began to increase at about $R_{\lambda }∼2$ from the Gaussian state and grew rapidly at $R_{\lambda }>20$ in the turbulent state. A contour map of the probability density functions (PDFs) indicated that PDF expansion of the gradients of the passive vector and passive scalar begins at around $R_{\lambda }\approx 4$, whereas that of the longitudinal velocity gradient PDF is more gradual. The left tails of the dissipation rate PDF were found to follow a power law with an exponent of 3/2 for the incompressible velocity and passive vector dissipation rates, and 1/2 for the scalar dissipation rate and the enstrophy; they remained constant for all Reynolds numbers, indicating the universality of the left tail. The analytical PDFs of the dissipation rates and enstrophy of the Gaussian state were obtained and found to be the Gamma distribution. It was shown that the number of terms contributing to the dissipation rates and the enstrophy determines the decay rates of the two PDFs for low to moderate amplitudes.

This article is part of the theme issue ‘Scaling the turbulence edifice (part 1)’.

## 1. Introduction

A peculiar characteristic of turbulence is that the statistical behaviour of the velocity field changes as spatial and temporal scales decrease [1]. At smaller scales, strong fluctuations occur more frequently. At very high Reynolds numbers, probability density functions (PDFs) of the velocity increment measured at two points separated by a distance of r are nearly Gaussian at larger distances; however, their PDF tails become longer as the distance decreases and the PDF of the velocity gradient becomes highly non-Gaussian [2–5]. A long tail indicates that extreme events occur more frequently in the turbulent than Gaussian state. However, the strength of these fluctuations and the likelihood of occurrence depend on the Reynolds number. These phenomena raise questions about the timing of the start of the turbulent state and the rate at which turbulence grows with the Reynolds number.

At low Reynolds numbers, flow is strongly affected by boundary conditions, even at small scales, such that a universal theory may not be feasible. However, for a thorough understanding of turbulence, it is necessary to know when it begins and how rapidly it develops. Yakhot & Donzis [6,7] considered this problem from a statistical perspective. They studied the variation of normalized moments of the velocity gradients (structure functions) for homogeneous isotropic turbulence (HIT) that is excited by a Gaussian random force with zero time correlation. This is an idealized set up but convenient for addressing this problem. At small Reynolds numbers, a smooth, laminar, but random flow field is generated, which obeys Gaussian statistics (Gaussian state). As the Reynolds number increases, the structure functions $S_{2n}$ of the velocity gradient begin to increase from the Gaussian value (2n−1)!! at about $R_{\lambda }\approx 9$ and grow monotonically, following a power law $R_{\lambda }^{d_{n}}$ (turbulent state). They found that the exponents $d_{n}$ were related to the scaling exponents $\xi_{n}$ of the velocity increments in the inertial range. Thus, scaling exponents $\xi_{n}$ were embedded within the exponent $d_{n}$s for structure functions of the velocity gradients at low Reynolds numbers. Schumacher *et al.* [8] analysed the Rayleigh Bénard convection problem from the perspective of a universal transition point and power law growth; they found that the structure functions did not grow monotonically from the Gaussian state to the turbulent state. Instead, once a minimum was attained, the structure functions increased as a power law with the same exponent as $d_{n}$ for the HIT case.

Another canonical problem in turbulence research is that of the scalar passively convected by turbulent flow [9]. Passive scalar fluctuations are well known to be more intermittent than velocity fluctuations, and the scaling exponents of the scalar moment in the inertial convective range are smaller, less universal, and saturate at high order than those of the velocity [9–12]. To explore the physics behind the differences between velocity and passive scalar fluctuations, Yang *et al.* [13,14] studied the statistical properties of an incompressible passive vector w and a passive scalar convected by the same turbulence. The visualization results showed that the pseudo-enstrophy ${\left(\nabla \times w\right)}^{2}$ of the passive vector was nearly sheetlike, similar to the case of the passive scalar, whereas the enstrophy was tubular. The scaling exponents of the moments of passive vector increments were found to be anomalous and non-universal at high order, similar to the passive scalar. It was argued that the differences in fluctuations between the turbulent velocity and passively convected fields depend on whether the fundamental equation is linear or nonlinear. Thus, strong fluctuations of passive fields at high Reynolds numbers occur due to the passive dynamics of fields obeying the linear fundamental equation(s). If these findings also apply at small Reynolds numbers, then transition of the passive scalar and vector may occur earlier or differently from the velocity, by sensitively responding to velocity field changes. Donzis *et al.* [15] studied this problem and found that the transition Reynolds number depends on the Schmidt number. Yasuda *et al.* [16] found that the skewness and flatness of the passive scalar under the mean scalar gradient showed sharp transitions at $P_{\lambda }\approx 2$, where $P_{\lambda }=u^{\prime }\lambda_{\theta }/\kappa$ is the micro scale Péclet number defined in terms of the Taylor micro scale $\lambda_{\theta }$ for the scalar.

In this study, we examined changes in fluctuation statistics from the Gaussian state to the turbulent state from the perspective of overall statistical properties, rather than focusing on the moments alone. We explored the statistical properties starting from low Reynolds numbers and the dissipation range towards high Reynolds numbers and the inertial range. Because the multivariate Gaussian random field is well known and easy to handle analytically, we were able to explore how the statistical laws governing these fluctuations change as the Reynolds number increases from low to moderate values, assuming that the statistical properties change progressively and not abruptly. Thus, we expected that extrapolation from the Gaussian state would provide more reliable clues with which to explore the turbulent state at high Reynolds numbers. Therefore, we considered the transition of fluctuations from the Gaussian state from a broader perspective, comparing velocities, passive vectors, and passive scalars, and examining spectra, dissipation, and PDFs. By comparing the three fields, we aimed to uncover more physics behind the fluctuation property differences. In particular, we focused on the PDF variation of the dissipation rates with respect to the Reynolds number, because these dissipation rates are directly related to the transfer rate of the excitation due to the nonlinear (convective) term and the singularity of the field, and because they are scalars computed as sums of several terms, reflecting spatial dimension effects. Also, the PDFs of the enstrophy were studied in comparison with the dissipation PDFs.

The paper is organized as follows. In §2, we describe the fundamental equations and numerical methods. In §3, we present the spectral scaling, and in §4, we present the moments. The PDF variation is examined in §5 and the PDFs of the dissipation and enstrophy are discussed in §6. Section 7 presents the conclusion.

## 2. Governing equations

We considered a passive incompressible vector and passive scalar convected by HIT in an incompressible fluid. The velocity u(x,t), convected passive vector w(x,t), and passive scalar θ(x,t) are governed by the following equations:

2.1 $$\begin{array}{rl} \left(\frac{\partial }{\partial t}+u\cdot \nabla \right)u & \;=-\nabla p+\nu \nabla^{2}u+f^{u},\;\nabla \cdot u=0, \end{array}$$

2.2 $$\begin{array}{rl} \left(\frac{\partial }{\partial t}+u\cdot \nabla \right)w & \;=-\nabla q+\alpha \nabla^{2}w+f^{w},\;\nabla \cdot w=0 \end{array}$$

2.3 $$\begin{array}{rl} \;\text{and}\;\left(\frac{\partial }{\partial t}+u\cdot \nabla \right)\theta & \;=\kappa \nabla^{2}\theta +f^{\theta }, \end{array}$$

where ν, α and κ are the kinetic viscosity and diffusive coefficients of the passive vector and passive scalar, respectively; p(x,t) is the reduced pressure divided by density; q(x,t) is the pseudo-pressure, ensuring the incompressibility of the passive vector. The external force $f^{u}$, injections $f^{w}$ and $f^{\theta }$ are assumed to be mutually independent, to obey Gaussian statistics with means of zero, and to change rapidly in time

2.4 $$\begin{array}{rl} \left\langle f_{i}^{u}(k,t)f_{j}^{u}(-k,s)\right\rangle & \;=\frac{1}{2}P_{ij}(k)\frac{F_{u}(k)}{2\pi k^{2}}\delta (t-s), \end{array}$$

2.5 $$\begin{array}{rl} \left\langle f_{i}^{w}(k,t)f_{j}^{w}(-k,s)\right\rangle & \;=\frac{1}{2}P_{ij}(k)\frac{F_{w}(k)}{2\pi k^{2}}\delta (t-s) \end{array}$$

2.6 $$\begin{array}{rl} \;\text{and}\;\left\langle f^{\theta }(k,t)f^{\theta }(-k,s)\right\rangle & \;=\frac{F_{\theta }(k)}{2\pi k^{2}}\delta (t-s), \end{array}$$

respectively, where $P_{ij}(k)=\delta_{ij}-k_{i}k_{j}/k^{2}$. The forcing is white noise and its spectrum is specified as follows:

2.7 $$F_{A}(k)=\left\{ \begin{array}{ll} \frac{\epsilon_{\text{in}}^{A}}{k_{\text{high}}-k_{\text{low}}} & \text{for}\;k_{\text{low}}\leq k\leq k_{\text{high}}\\ 0 & \text{otherwise} \end{array}\right.,$$

where A denotes u,w, and θ, and $k_{\text{low}}=2,k_{\text{high}}=3$ for all Runs. The injection rate for $\epsilon_{\text{in}}^{\theta }$ is selected to be equal to one component among u or w, as follows:

2.8 $$\epsilon_{\text{in}}^{u,w}=\int_{0}^{\infty }F_{u,w}(k)\;\text{d}k\;\text{and}\;\epsilon_{\text{in}}^{\theta }=\int_{0}^{\infty }F_{\theta }(k)\;\text{d}k=\frac{1}{3}\epsilon_{\text{in}}^{u,w}.$$

As numerical methods, we used the pseudo-spectral method for space and the second-order Runge–Kutta method for advancing the time. The computation was started with initially Gaussian random fields with means of zero and the spectrum that is defined as follows:

2.9 $$E_{A}(k,0)=N_{A}u_{0}^{2}k_{0}^{-5}k^{4}\exp\left(-2{\left(\frac{k}{k_{0}}\right)}^{2}\right),$$

where $k_{0}=2$ and $N_{A}$ are normalization constants, such that the total turbulent energy is equal to $1.5\times 10^{-2}$ for A=u,w,θ.

The fundamental statistics at low order and intermittency of the above three fields at moderate Reynolds numbers were described in detail previously in [13,14]. To explore the statistical changes with increasing Reynolds numbers, we performed direct numerical simulations (DNSs). The fundamental turbulence and numerical parameters used in various simulation runs are listed in table 1. ${\bar{\epsilon }}_{u,w\;\text{and}\;\theta }$ are the mean dissipation rates of the kinetic energy, the pseudo-kinetic energy, and the scalar variance per unit mass. $\bar{\eta }$ is the mean Kolmogorov length, $\lambda_{u,w\;\text{and}\;\theta }$ are the Taylor microscale, and $L_{u,w\;\text{and}\;\theta }$ are the integral scale. The Schmidt numbers $Sc_{w}=\nu /\alpha$ and $Sc_{\theta }=\nu /\kappa$ were set at unity for all DNSs. Run label 128A indicates Run A, with $128^{3}$ grid points. Initially, transient parts of the dataset were discarded, and a very long average time duration in a statistically steady state was set to obtain well-converged statistics. To ensure sufficient spatial resolution, $K_{\text{max}}\bar{\eta }$ was set at ≥3.5 as previously reported [17].

**Table 1.** Run label number denotes the number of grid points, and capital letters indicate series of runs with increasing Reynolds numbers. ν kinematic viscosity, $R_{\lambda }$ Taylor macroscale Reynolds number, $K_{\text{max}}\eta$ maximum wavenumber in Kolmogorov units, $T_{\text{av}}/T_{E}$ average time duration in large-eddy turnover time units, $\epsilon_{u,w,\theta }$ mean dissipation rates, $\lambda_{u,w,\theta }$ Taylor microscale, $L_{u,w,\theta }$ integral scale.

run	ν	$R_{\lambda }$	$K_{\text{max}}\bar{\eta }$	$T_{\text{av}}/T_{E}$	${\bar{\epsilon }}_{u}$	${\bar{\epsilon }}_{w}$	${\bar{\epsilon }}_{\theta }$	$\lambda_{u}$	$\lambda_{w}$	$\lambda_{\theta }$	$L_{u}$	$L_{w}$	$L_{\theta }$
128A	2.0	0.13	61	15	2.70	2.68	0.892	0.920	0.919	1.01	1.09	1.09	0.728
128B	1.0	0.36	47	28	2.68	2.69	0.904	0.919	0.918	1.01	1.09	1.09	0.730
128C	0.7	0.61	34	34	2.70	2.70	0.905	0.919	0.917	0.731	1.09	1.09	0.731
128D	0.5	1.0	28	250	2.69	2.70	0.899	0.918	0.916	0.734	1.10	1.10	0.734
128E	0.3	2.1	19	317	2.69	2.69	0.895	0.912	0.902	0.986	1.11	1.10	0.741
128F	0.2	3.8	14	377	2.70	2.71	0.902	0.898	0.871	0.948	1.13	1.10	0.742
256A	0.14	6.2	22	104	2.72	2.74	0.913	0.870	0.824	0.890	1.14	1.09	0.733
256B	0.1	9.2	17	138	2.72	2.70	0.892	0.827	0.757	0.812	1.13	1.06	0.705
256C	0.07	13	13	135	2.71	2.70	0.905	0.764	0.679	0.734	1.10	1.02	0.674
512A	0.04	23	17	135	2.70	2.68	0.893	0.652	0.553	0.581	1.03	0.950	0.621
512B	0.025	32	12	156	2.72	2.76	0.911	0.548	0.453	0.472	0.948	0.893	0.578
512C	0.016	44	8.4	176	2.74	2.67	0.894	0.454	0.370	0.381	0.875	0.842	0.543
512D	0.01	58	5.9	193	2.78	2.75	0.893	0.366	0.296	0.302	0.816	0.803	0.516
512E	0.008	66	5.0	201	2.79	2.80	0.942	0.329	0.266	0.269	0.792	0.789	0.505
512F	0.005	84	3.5	256	2.83	2.80	0.928	0.261	0.212	0.211	0.750	0.771	0.495
1024A	0.003	110	4.8	12.2	2.83	2.84	0.916	0.204	0.166	0.165	0.725	0.748	0.493

## 3. Spectra

The wavenumber spectra are defined by

3.1 $$\frac{1}{2}\left\langle u^{2}\right\rangle =\int_{0}^{\infty }E_{u}(k)\;\text{d}k,\;\frac{1}{2}\left\langle w^{2}\right\rangle =\int_{0}^{\infty }E_{w}(k)\;\text{d}k\;\text{and}\;\frac{1}{2}\left\langle \theta^{2}\right\rangle =\int_{0}^{\infty }E_{\theta }(k)\;\text{d}k,$$

respectively, and are normalized by the Kolmogorov variables for $Sc_{w}=Sc_{\theta }=1$ as

3.2 $$\begin{array}{rl} E_{u}(k) & \;={\bar{\epsilon }}_{u}^{1/4}\nu^{5/4}f(k\bar{\eta }), \end{array}$$

3.3 $$\begin{array}{rl} E_{w}(k) & \;={\bar{\epsilon }}_{w}{\left(\frac{{\bar{\epsilon }}_{u}}{\nu }\right)}^{-1/2}\bar{\eta }\;f_{w}(k\bar{\eta }) \end{array}$$

**Figure 1.**
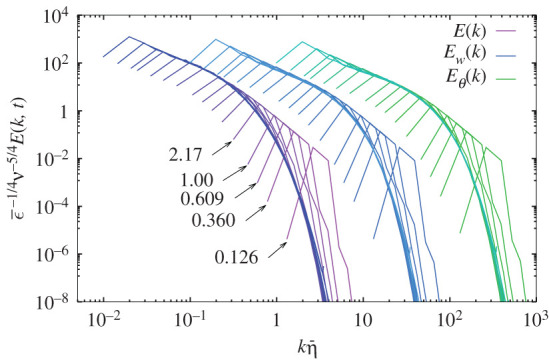
Variation of normalized spectra against $R_{\lambda }$. Numbers indicate $R_{\lambda }$ for Runs 128A-128E, and the longest curve corresponds to Run 1024A at $R_{\lambda }=110$. Magenta-blue curves indicate $\nu^{-5/4}{\bar{\epsilon }}^{-1/4}E(k)$, sky blue curves are for ${\bar{\epsilon }}_{w}{\left(\nu /{\bar{\epsilon }}_{u}\right)}^{1/2}{\bar{\eta }}^{-1}E_{w}(k)$, and green for ${\bar{\epsilon }}_{\theta }{\left(\nu /{\bar{\epsilon }}_{u}\right)}^{1/2}{\bar{\eta }}^{-1}E_{\theta }(k)$, respectively. Curves for $E_{w}(k)$ and $E_{\theta }(k)$ are shifted horizontally and vertically by (10,100) and $\left(100,10^{4}\right)$, respectively. (Online version in colour.)

3.4 $$\begin{array}{rl} \;\text{and}\;E_{\theta }(k) & \;={\bar{\epsilon }}_{\theta }{\left(\frac{{\bar{\epsilon }}_{u}}{\nu }\right)}^{-1/2}\bar{\eta }\;f_{\theta }(k\bar{\eta }), \end{array}$$

where $\eta ={\left(\nu^{3}/{\bar{\epsilon }}_{u}\right)}^{1/4}$ and $f(x),f_{w}(x)$ and $f_{\theta }(x)$ are universal non-dimensional functions. Figure 1 shows the variation of three spectra with increasing Reynolds numbers. The curves for $f_{w}(k\bar{\eta })$ and $f_{\theta }(k\bar{\eta })$ are shifted horizontally and vertically by (10,100) and $\left(100,10^{4}\right)$, respectively, for easier viewing.

The functional forms of the three spectra change in similar ways, extending toward low wavenumbers as the Reynolds number increases. When $R_{\lambda }<2$, the spectral curves do not collapse, but tend to merge on a single curve for $R_{\lambda }>2$, such that the scaling of spectra in terms of units in the Kolmogorov–Obukhov–Corrsin theory begins to work when $R_{\lambda }>2$. The normalized spectra extend to a lower wavenumber range and approach $k^{-5/3}$ behaviour within a very narrow range in Run 1024A.

One measure of the transfer of kinetic energy, pseudo-kinetic energy, and scalar variance to small scales is skewness, which is defined by the equation of motion as follows [13,18]:

3.5 $$\begin{array}{rl} S_{K}^{u} & \;=\frac{\left\langle {\left(\partial u_{1}/\partial x_{1}\right)}^{3}\right\rangle }{{\left\langle {\left(\partial u_{1}/\partial x_{1}\right)}^{2}\right\rangle }^{3/2}}, \end{array}$$

3.6 $$\begin{array}{rl} S_{K}^{w} & \;=\frac{\left\langle (\partial u_{1}/\partial x_{1})[{\left(\partial w_{1}/\partial x_{1}\right)}^{2}+{\left(\partial w_{2}/\partial x_{1}\right)}^{2}+{\left(\partial w_{3}/\partial x_{1}\right)}^{2}]\right\rangle }{{\left\langle {\left(\partial u_{1}/\partial x_{1}\right)}^{2}\right\rangle }^{1/2}\left\langle {\left(\partial w_{1}/\partial x_{1}\right)}^{2}+{\left(\partial w_{2}/\partial x_{1}\right)}^{2}+{\left(\partial w_{3}/\partial x_{1}\right)}^{2}\right\rangle }, \end{array}$$

**Figure 2.**
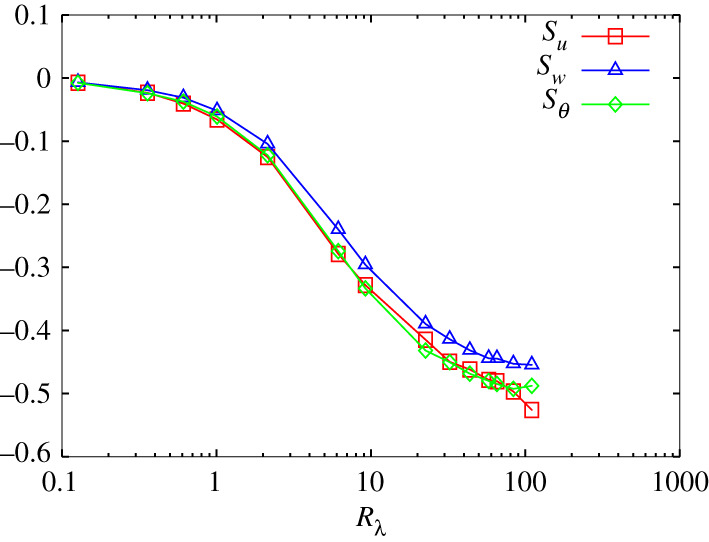
Variation in skewness with Reynolds number. Red, blue and green curves with symbol indicate $S_{K}^{u}$, $S_{K}^{w}$ and $S_{K}^{\theta }$, respectively. (Online version in colour.)

3.7 $$\begin{array}{rl} \;\text{and}\;S_{K}^{\theta } & \;=\frac{\left\langle (\partial u_{1}/\partial x_{1}){\left(\partial \theta /\partial x_{1}\right)}^{2}\right\rangle }{{\left\langle {\left(\partial u_{1}/\partial x_{1}\right)}^{2}\right\rangle }^{1/2}\left\langle {\left(\partial \theta /\partial x_{1}\right)}^{2}\right\rangle }, \end{array}$$

respectively. Figure 2 shows the variation in skewness with the Reynolds number. All $S_{K}^{A}$ are nearly zero at $R_{\lambda }=0.13$, slowly decrease to $R_{\lambda }\approx 1$, quickly decrease for $2<R_{\lambda }<20$, and finally approach $S_{K}^{w}\approx -0.45$ and $S_{K}^{u}\approx S_{K}^{\theta }\approx -0.5$. This result indicates that the transfer of (pseudo) kinetic energy and scalar variance begins at $R_{\lambda }\approx 2$, which is consistent with the observation that the wavenumber spectra begin to collapse in the context of Kolmogorov–Obukhov–Corrsin scaling.

## 4. Moments

### (a) Gradient field structure functions

Next, we considered the high-order statistics of the gradient fields. The structure functions of the longitudinal and transverse derivatives of the velocity, passive vector, and passive scalar were defined as follows:

4.1 $$\begin{array}{rl} S_{2n}^{u_{L}} & \;=\frac{\left\langle {\left(\partial u_{1}/\partial x_{1}\right)}^{2n}\right\rangle }{{\left\langle {\left(\partial u_{1}/\partial x_{1}\right)}^{2}\right\rangle }^{n}}, \end{array}$$

4.2 $$\begin{array}{rl} S_{2n}^{u_{T}} & \;=\frac{\left\langle {\left(\partial u_{1}/\partial x_{2}\right)}^{2n}\right\rangle }{{\left\langle {\left(\partial u_{1}/\partial x_{2}\right)}^{2}\right\rangle }^{n}}, \end{array}$$

4.3 $$\begin{array}{rl} S_{2n}^{w_{L}} & \;=\frac{\left\langle {\left(\partial w_{1}/\partial x_{1}\right)}^{2n}\right\rangle }{{\left\langle {\left(\partial w_{1}/\partial x_{1}\right)}^{2}\right\rangle }^{n}}, \end{array}$$

4.4 $$\begin{array}{rl} S_{2n}^{w_{T}} & \;=\frac{\left\langle {\left(\partial w_{1}/\partial x_{2}\right)}^{2n}\right\rangle }{{\left\langle {\left(\partial w_{1}/\partial x_{2}\right)}^{2}\right\rangle }^{n}} \end{array}$$

4.5 $$\begin{array}{rl} \;\text{and}\;S_{2n}^{\theta } & \;=\frac{\left\langle {\left(\partial \theta /\partial x_{1}\right)}^{2n}\right\rangle }{{\left\langle {\left(\partial \theta /\partial x_{1}\right)}^{2}\right\rangle }^{n}}. \end{array}$$

For the multivariate Gaussian random field,

4.6 $$S_{2n}^{G}=(2n-1)!!.$$

**Figure 3.**
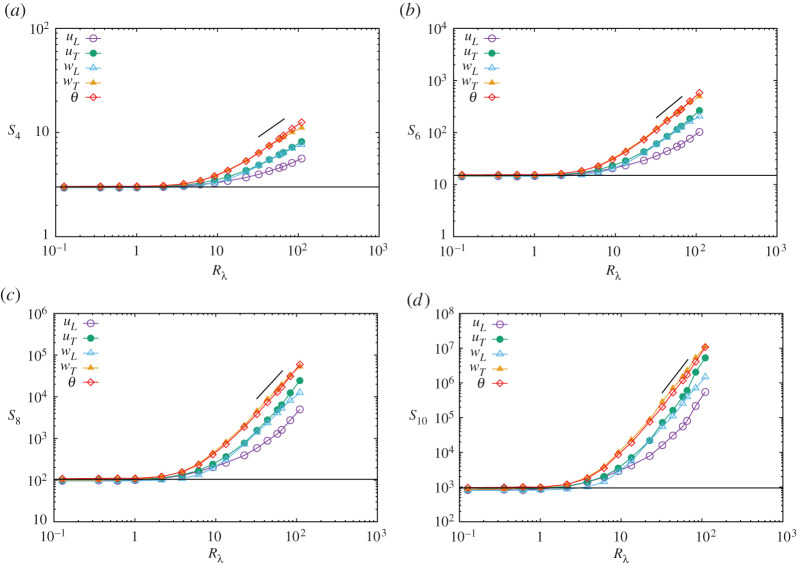
Variation of structure functions with Reynolds number. (*a*) $S_{4}^{A}$, (*b*) $S_{6}^{A}$, (*c*) $S_{8}^{A}$, (*d*) $S_{10}^{A}$. A denotes $u_{L},u_{T},w_{L},w_{T},\theta$. Horizontal line indicates Gaussian values at $S_{2n}^{G}=(2n-1)!!$. Straight line indicates the slope and range computed for $S_{n}^{\theta }$ for n=2,4,6,8,10. (Online version in colour.)

Figure 3 shows the variation in $S_{p}^{A}$ for p=4,6,8,10 with the Reynolds number. We observed the following: (1) When $R_{\lambda }<2$, all curves are on the Gaussian values, and begin to increase at around $R_{\lambda ,4}\approx 4$ for $S_{4}^{A}$ and $R_{\lambda ,10}\approx 2$ for $S_{10}^{A}$, irrespective of the moment type, indicating that the transition $R_{\lambda }^{\text{tr}}$ slowly decreases with the moment order, consistent with a previous report [6]. (2) For $30<R_{\lambda }<70$, $S_{2n}^{A}$ for $A=u_{T},w_{L},w_{T},\theta$ increases approximately in line with the quasi power law $R_{\lambda }^{\rho_{2n}^{A}}$, whereas, $S_{2n}^{u_{L}}$ increases the most slowly and remains smallest for all values of $R_{\lambda }$. For larger Reynolds numbers, $S_{2n}^{u_{L}}$ appears to increase faster; however, the growth trend remains uncertain due to considerable scatter among the data points. (3) For $30<R_{\lambda }<70$, the moment values for a given order are in the order of

4.7 $$S_{2n}^{u_{L}}<S_{2n}^{u_{T}}∼S_{2n}^{w_{L}}<S_{2n}^{w_{T}}∼S_{2n}^{\theta }.$$

This property is consistent with the observation that the scaling exponents of the structure functions in the inertial-convective range are ordered in the same manner as equation (4.7) [14]. (4) Although the curves show larger scatter for high-order moments and large Reynolds numbers, and the range of $R_{\lambda }$ is narrow, it is interesting to measure the exponents, which are defined as

4.8 $$S_{n}^{A}\propto R_{\lambda }^{\rho_{n}^{A}},$$

for $A=u_{L},u_{T},w_{L},w_{t},\theta$, as listed in table 2. The exponents vary considerably depending on the range of $R_{\lambda }$ (figure 3); therefore, we cite the range of $R_{\lambda }$ in table 2 and the exponents without error bars, which should be interpreted as a reference only. The exponent $\rho_{n}^{u_{L}}$ is smaller than other exponents for the Reynolds numbers studied.

**Table 2.** Power law exponents of the structure functions of gradients $S_{n}\propto R_{\lambda }^{\rho_{n}^{A}}$ and dissipation rate moments $M_{n}\propto R_{\lambda }^{\xi_{n}^{B}}$. The range is the domain of $R_{\lambda }$ for exponent computation.

field	range	n=2	n=3	n=4	n=5
$\rho_{2n}^{u_{L}}$	[30, 70]	0.246	0.722	1.41	2.26
$\rho_{2n}^{u_{T}}$	[30, 70]	0.395	1.09	1.99	3.02
$\rho_{2n}^{w_{L}}$	[30, 70]	0.404	1.07	1.87	2.80
$\rho_{2n}^{w_{T}}$	[30, 70]	0.478	1.20	2.02	2.89
$\rho_{2n}^{\theta }$	[30, 70]	0.543	1.28	2.11	3.01
$\xi_{n}^{\epsilon_{u}}$	[70, 120]	0.343	1.07	2.23	3.64
$\xi_{n}^{\epsilon_{w}}$	[20, 90]	0.400	1.08	1.90	2.79
$\xi_{n}^{\epsilon_{\theta }}$	[20, 90]	0.529	1.26	2.07	2.93

Previously, we reported local scaling exponents as follows [3,12]: $\left\langle |\delta u_{L}(r){|}^{n}\right\rangle \propto r^{\zeta_{n}^{L}(r)}$, $\left\langle |\delta u_{T}(r){|}^{n}\right\rangle \propto r^{\zeta_{n}^{T}(r)}$ and $\left\langle |\delta \theta (r){|}^{n}\right\rangle \propto r^{\zeta_{n}^{\theta }(r)}$ in the inertial range and in the order of

4.9 $$\zeta_{n}^{\theta }<\zeta_{n}^{T}<\zeta_{n}^{L},\;\text{for}\;n\geq 4.$$

We also observed that the crossover lengths $l_{n,\text{cr}}^{u_{T}}$ and $l_{n,\text{cr}}^{\theta }$ of the local scaling exponents of $\left\langle |\delta u_{T}(r){|}^{n}\right\rangle \propto r^{\zeta_{n}^{T}(r)}$ and $\left\langle |\delta \theta (r){|}^{n}\right\rangle \propto r^{\zeta_{n}^{\theta }(r)}$, respectively, which were defined as the transition length from the inertial (-convective) range value to the dissipation range value, were the same as (and on the same order as) $r/\bar{\eta }\approx 50$, and were insensitive to the moment order n. However, the crossover length $l_{n,\text{cr}}^{u_{L}}$ for $\left\langle |\delta u_{L}(r){|}^{n}\right\rangle \propto r^{\zeta_{n}^{L}(r)}$ is longer than $l_{n,\text{cr}}^{u_{T}}$ and slowly increases with n. Thus, we can estimate the moments of the gradient using the following formula: $\left\langle {\left(\nabla \phi \right)}^{2n}\right\rangle \approx \left\langle |\delta \phi {|}^{2n}\right\rangle /{\left(l_{n,\text{cr}}^{\phi }\right)}^{2n}$, where ϕ represents $u_{T}$ or θ; this estimate is consistent with observations (2) and (3). Applying the crossover length $l_{n,\text{cr}}^{\phi }$ to estimate the gradient is not trivial, and the use of different length scales such as the order-dependent Kolmogorov length $\eta_{n}$, which decreases with the order n, may lead to different solutions; however, we will not discuss this point further. The similar behaviour of w and θ is explained by their obeying linear equations and being passively advected by turbulent velocity that is governed by the Navier–Stokes equations, which are nonlinear. This case differs from that of Rayleigh–Bérnard convection, in which temperature acts on the velocity field through the buoyancy force [5,8].

### (b) Dissipation rate moments

The dissipation rates are defined as follows:

4.10 $$\begin{array}{rl} {\tilde{\epsilon }}_{u}(x,t) & \;=\frac{1}{2}\nu {\left(\frac{\partial u_{i}}{\partial x_{j}}+\frac{\partial u_{j}}{\partial x_{i}}\right)}^{2}, \end{array}$$

4.11 $$\begin{array}{rl} {\tilde{\epsilon }}_{w}(x,t) & \;=\frac{1}{2}\alpha {\left(\frac{\partial w_{i}}{\partial x_{j}}+\frac{\partial w_{j}}{\partial x_{i}}\right)}^{2} \end{array}$$

**Figure 4.**
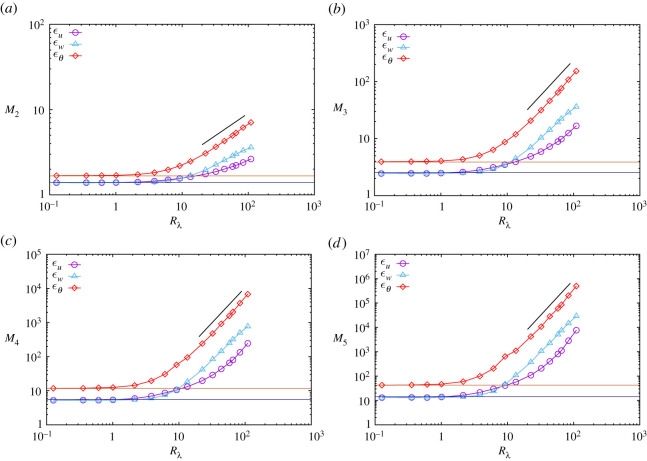
Variation of the moments of dissipation rates with Reynolds number. (*a*) $M_{2}^{A}$, (*b*) $M_{3}^{A}$, (*c*) $M_{4}^{A}$, (*d*) $M_{5}^{A}$. A denotes $\epsilon_{u},\epsilon_{w}\;\text{and}\;\epsilon_{\theta }$. The straight line indicates the slope and range computed for $M_{n}^{\epsilon_{\theta }}$ for n=2,3,4,5. Horizontal lines indicate the moments of the dissipation rates corresponding to Gaussian random fields. (Online version in colour.)

4.12 $$\begin{array}{rl} \;\text{and}\;{\tilde{\epsilon }}_{\theta }(x,t) & \;=\kappa {\left(\frac{\partial \theta }{\partial x_{j}}\right)}^{2}, \end{array}$$

where summation over repeated indices is assumed. The dissipation rate moments are normalized by the mean value as follows:

4.13 $$M_{n}^{A}=\left\langle {\left(\frac{{\tilde{\epsilon }}_{A}}{{\bar{\epsilon }}_{A}}\right)}^{n}\right\rangle =\left\langle \epsilon_{A}^{n}\right\rangle \;\text{and}\;\epsilon_{A}\equiv \frac{{\tilde{\epsilon }}_{A}}{{\bar{\epsilon }}_{A}},$$

where A represents u,w and θ and ${\bar{\epsilon }}_{A}=\left\langle {\tilde{\epsilon }}_{A}\right\rangle$ is the mean dissipation rate. The variation of the moments with the Reynolds number is shown in figure 4. At very low Reynolds numbers, $M_{n}^{A}$ values are the same as those of the Gaussian field (see next section) and independent of $R_{\lambda }$, whereas $M_{n}^{\theta }$ differs from $M_{n}^{u}=M_{n}^{w}$. The moments begin to increase at around $R_{\lambda }∼4$ for $M_{2}^{A}$ and $R_{\lambda }∼2$ for $M_{5}^{A}$. The data scatter is weaker than that of $S_{2n}^{A}$ because the dissipation rate is computed as the sum of five terms for $\epsilon_{u}$ and $\epsilon_{w}$, and of three terms for $\epsilon_{\theta }$. $M_{n}^{u}$ increases slowly, whereas $M_{n}^{w}$ and $M_{n}^{\theta }$ rapidly establish power law growth in $R_{\lambda }$, as follows:

4.14 $$M_{n}^{A}\propto R_{\lambda }^{\xi_{n}^{A}}.$$

The computed exponents are listed in table 2. In this study, when $\text{Re}\propto R_{\lambda }^{2}$ was counted, $\xi_{n}^{u}$ values were slightly larger than those of [6,17,19], partly due to the narrow range of $70\leq R_{\lambda }\leq 120$ in this study.

## 5. PDFs

**Figure 5.**
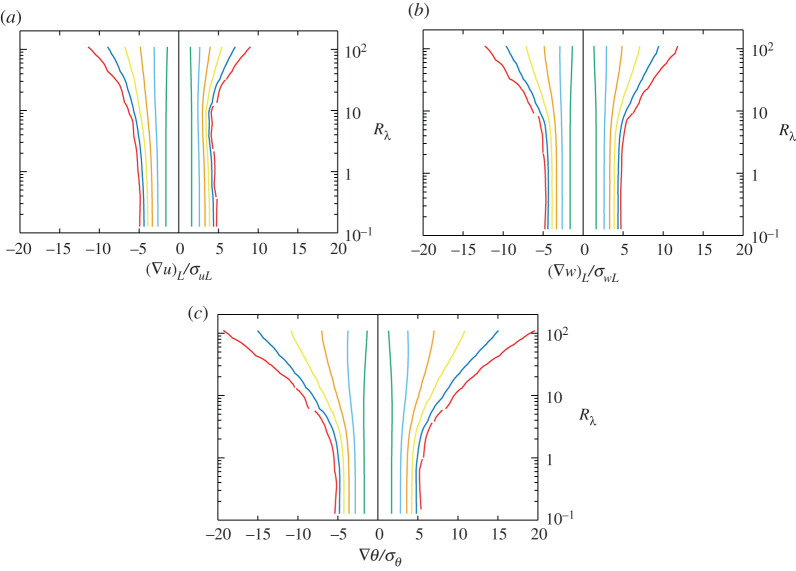
Contour map of PDFs. (*a*) $\bar{P}(\nabla_{L}u)$, (*b*) $\bar{P}(\nabla_{L}w)$ and (*c*) $\bar{P}(\nabla \theta )$. Lines are plotted every decade from the innermost curve of $10^{-1}$ to the outermost curve of $10^{-6}$. (Online version in colour.)

**Figure 6.**
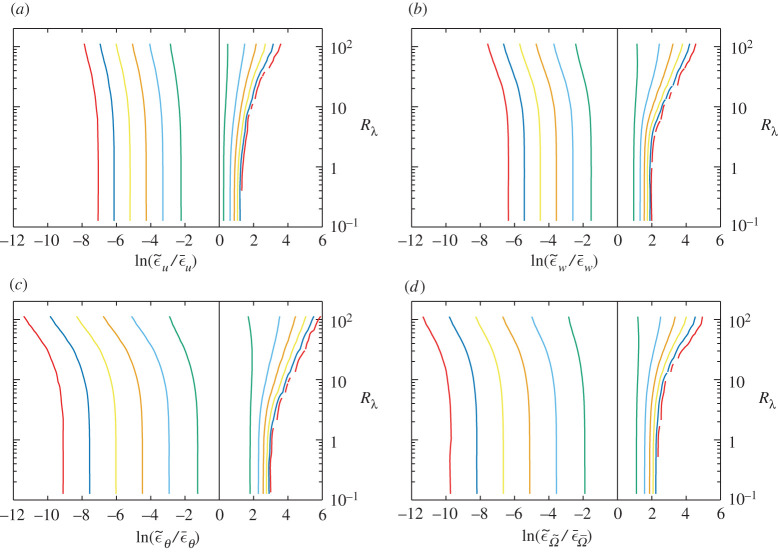
Contour map of PDFs. (*a*) $\bar{P}(\ln(\epsilon_{u}))$, (*b*) $\bar{P}(\ln(\epsilon_{w}))$, (*c*) $\bar{P}(\ln(\epsilon_{\theta }))$ and (*d*) $\bar{P}(\ln(\Omega ))$. Lines are plotted every decade from the innermost curve of $10^{-1}$ to the outermost curve of $10^{-6}$. (Online version in colour.)

PDFs indicate the overall fluctuation, from weak to extreme events. To examine changes in the PDFs as the Reynolds number increased, we created a contour map of PDFs in the space of amplitude and Reynolds numbers in figure 5 for the gradients, and those in figure 6 for the dissipation rates and enstrophy. Curves were plotted for every decade, from the innermost curve, corresponding to $10^{-1}$, to the outermost curve, corresponding to $10^{-6}$. In figure 5, when the Reynolds number was <1, the contour lines were parallel in the vertical direction and almost symmetric with respect to the zero amplitude line. The outermost contour lines began to expand at about $R_{\lambda }=4$. Expansion gradually increased with the Reynolds number, such that the expansion rate was higher for the outer curves, whereas the innermost curves for all PDFs tended to shrink toward zero amplitude at larger Reynolds numbers, and PDFs near the peak became steeper as $R_{\lambda }$ increased. The contour lines for $\bar{P}(\partial u_{1}/\partial x_{1})$ were asymmetric, reflecting the kinetic energy transfer to smaller scales, whereas $\bar{P}(\partial u_{1}/\partial x_{2})$, $\bar{P}(\partial w_{1}/\partial x_{1})$, $\bar{P}(\partial w_{1}/\partial x_{2})$ and $\bar{P}(\partial \theta /\partial x_{2})$ were nearly symmetric. The speed of expansion was on the order of $\bar{P}(\partial w_{1}/\partial x_{1})<\bar{P}(\partial u_{1}/\partial x_{2})<\bar{P}(\partial w_{1}/\partial x_{2})∼\bar{P}(\partial \theta /\partial x_{2})$ (figures $\bar{P}(\partial u_{1}/\partial x_{2})$ and $\bar{P}(\partial w_{1}/\partial x_{2})$ are not shown), which is consistent with the increasing trend of the moments. The right contour lines for $\bar{P}(\partial u_{1}/\partial x_{1})$ in figure 5*a* shrink once at $R_{\lambda }\approx 10$ and begin to expand at higher Reynolds numbers; this behaviour was not observed for the other PDFs. One possible explanation that we considered was insufficient statistical convergence of $\bar{P}(\partial u_{1}/\partial x_{1})$. However, we confirmed that extension of the average time duration did not influence this shrinkage; therefore, this phenomenon remains unexplained.

Figure 6 shows a plot of PDFs of the logarithm of the dissipation rate and of the enstrophy. Notably, when the Reynolds number increased, the contour lines of the left tail expanded, but remained parallel and equidistant for all Reynolds numbers examined in this study, indicating the power law behaviour of the PDFs at small amplitudes. By contrast, the contour lines of the right tail of the PDFs expanded progressively as the Reynolds number increased. In figure 6*a*, the rightmost curve begins to expand at around $R_{\lambda }\approx 4$, whereas in figure 6*c*, curve expansion begins at about $R_{\lambda }=1$ and $\bar{P}(\ln(\epsilon_{w}))$ lies between these cases. This observation is consistent with figure 5; however, the waviness of the curves makes the trend less clear. Although the enstrophy PDF is discussed in detail in the next section, it is seen in figure 6*d* that the transition of $\bar{P}(\ln(\Omega ))$ from the Gaussian state to the turbulent state occurs at around $R_{\lambda }\approx 8$, slightly slower than $\bar{P}(\ln(\tilde{\epsilon }))$. The enstrophy PDF is wider than the dissipation PDF and slightly narrower than the scalar dissipation. The transition from Gaussian to turbulent fluctuation is more apparent and occurs at lower Reynolds numbers for the passive scalar than for the velocity.

It is both important and useful to consider the PDF of the dissipation rate under a Gaussian random velocity field (see also [20]). As described in the appendix, the PDFs for this case are analytically derived and equivalent to the Gamma distribution

5.1 $${\bar{P}}^{G}(\epsilon_{A})\;\text{d}\epsilon_{A}=\frac{{\left(5/2\right)}^{5/2}}{\Gamma (5/2)}\;\epsilon_{A}^{3/2}\exp\left(-\frac{5}{2}\epsilon_{A}\right)\;\text{d}\epsilon_{A},\;\epsilon_{A}=\frac{{\tilde{\epsilon }}_{A}}{{\bar{\epsilon }}_{A}},\;A=u\;\text{or}\;w$$

and

5.2 $${\bar{P}}^{G}(\epsilon_{\theta })\;\text{d}\epsilon_{\theta }=\frac{{\left(3/2\right)}^{3/2}}{\Gamma (3/2)}\;\epsilon_{\theta }^{1/2}\exp\left(-\frac{3}{2}\epsilon_{\theta }\right)\;\text{d}\epsilon_{\theta },\;\epsilon_{\theta }=\frac{{\tilde{\epsilon }}_{\theta }}{{\bar{\epsilon }}_{\theta }}.$$

At small amplitudes, the PDFs follow a power law, with exponents 3/2 for ${\bar{P}}^{G}(\epsilon_{u})$ and ${\bar{P}}^{G}(\epsilon_{w})$, and 1/2 for ${\bar{P}}^{G}(\epsilon_{\theta })$. At large amplitudes, the PDFs are exponential, with decay rates of 5/2 for the velocity and passive vector, and 3/2 for the passive scalar. Thus, ${\bar{P}}^{G}(\epsilon_{u})$ and ${\bar{P}}^{G}(\epsilon_{w})$ decay faster than ${\bar{P}}^{G}(\epsilon_{\theta })$ at both small and large amplitudes. The moments of the dissipation rates are readily computed as follows:

5.3 $$\left\langle {\left(\frac{{\tilde{\epsilon }}_{u}}{{\bar{\epsilon }}_{u}}\right)}^{n}\right\rangle =\left\langle {\left(\frac{{\tilde{\epsilon }}_{w}}{{\bar{\epsilon }}_{w}}\right)}^{n}\right\rangle =\frac{1}{3}\frac{(2n+3)!!}{5^{n}}$$

and

5.4 $$\left\langle {\left(\frac{{\tilde{\epsilon }}_{\theta }}{{\bar{\epsilon }}_{\theta }}\right)}^{n}\right\rangle =\frac{(2n+1)!!}{3^{n}},$$

and are shown as horizontal lines in figure 4. The moments of the dissipation rates corresponding to the Gaussian field increase less slowly with order n than the structure functions of the gradients in equation (4.6).

**Figure 7.**
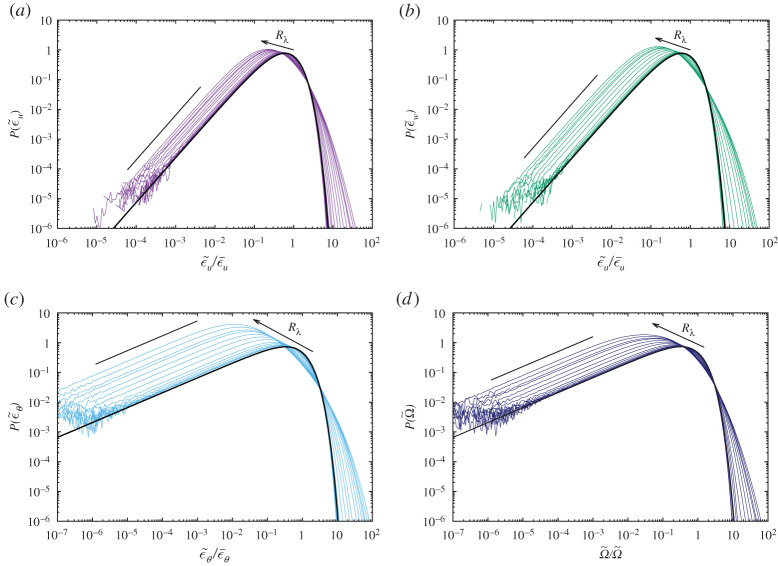
Variation of the PDFs of the dissipation rates and estrophy against $R_{\lambda }$. (*a*) $P({\tilde{\epsilon }}_{u})$, (*b*) $P({\tilde{\epsilon }}_{w})$, (*c*) $P({\tilde{\epsilon }}_{\theta })$ and (*d*) $P(\tilde{\Omega })$. The inner most curve is for $R_{\lambda }=0.13$ (Run 128A) and the outer most one is for $R_{\lambda }=110$ (Run 1024A). The arrow shows the direction of increase of $R_{\lambda }$ and the PDF curves gradually expand with increase of $R_{\lambda }$. Thick black curve indicates the PDFs of the dissipation rates for (*a*) to (*c*,*d*) enstrophy for the Gaussian random field. Straight lines indicate a slope of 3/2 for (*a*) $P({\tilde{\epsilon }}_{u})$ and (*b*) $P({\tilde{\epsilon }}_{w})$, and 1/2 for (*c*) $P({\tilde{\epsilon }}_{\theta })$ and (*d*) $P(\tilde{\Omega })$. (Online version in colour.)

Figure 7 shows the normalized PDFs of (*a*) $\epsilon_{u}$, (*b*) $\epsilon_{w}$, (*c*) $\epsilon_{\theta }$ and (*d*) Ω for all Reynolds numbers. The thick black line indicates the PDFs (5.1) for (*a*) and (*b*), and (5.2) for (*c*), and the straight line indicates the slope 3/2 for (*a*) and (*b*), and 1/2 for (*c*) and (*d*). We observe the following: (1) When $R_{\lambda }$ is small (Gaussian state), the DNS curves collapse well, following the Gamma distribution. (2) $\bar{P}(\epsilon_{\theta })$ is the widest among the three PDFs, and decays more slowly than the other PDFs. (3) The peaks of all PDFs shift toward lower and then higher amplitudes as $R_{\lambda }$ increases. (4) The left tails of all PDFs obey the same power law as that for the corresponding Gaussian random fields, irrespective of $R_{\lambda }$. (5) However, the right tails decrease rapidly, but more slowly than the exponential.

Careful inspection reveals that the right tails of $\bar{P}(\epsilon_{A})$ for small $R_{\lambda }<1$ decay slightly faster than the Gamma distribution, because the forcing range is at low wavenumbers and remains narrow ($2\leq k_{f}\leq 3$). Furthermore, the nonlinear interaction is negligible for $R_{\lambda }<1$, such that the molecular dissipation (Laplacian operator) acts more strongly to smear the gradient fields compared to the case of a simple Gaussian random field without any dynamics.

As shown in the appendix, the power law exponents $\xi_{A}$ and decay rates $C_{A}$ in the exponential of the PDF ${\bar{P}}^{G}(s)\propto x^{\xi_{A}}\exp(-C_{A}x)$ are determined by the number of terms contributing to $\epsilon_{A}$, $N_{u}(d)=N_{w}(d)=(d-1)(d+2)/2$ or $N_{\theta }(d)=d$, where d represents the spatial dimensions. In d=3, $\xi_{u}=N_{u}/2-1=3/2=\xi_{w}$ and $\xi_{\theta }=N_{\theta }/2-1=1/2$, whereas $C_{u}=C_{w}=5/2$ and $C_{\theta }=3/2$. Thus, $\bar{P}(\epsilon_{\theta })$ has longer left tails in the logarithmic plot and longer right tails than $\bar{P}(\epsilon_{u})$ and $\bar{P}(\epsilon_{w})$ for low to moderate Reynolds numbers in this study. The power law behaviour of the left tails of the dissipation rate PDFs is independent of the Reynolds number, and therefore universal. Indeed, the same power law was observed, with exponents (−7/2 for $P(\bar{\epsilon }/\tilde{\epsilon })$ and −5/2 for $\bar{P}(\bar{\Omega }/\tilde{\Omega })$) at $R_{\lambda }=240$ and 1000 in fig. 5*a* of [20].

## 6. Dissipation rate and enstrophy PDFs

The PDFs of the kinetic energy dissipation rate and enstrophy have been discussed in many studies, to determine the relative length of the tails and whether both moments increase following the same power law as the Reynolds number [17,20–26]. Donzis *et al.* [17] have found that the dissipation PDF is smaller than the enstrophy PDF at moderate amplitudes, but becomes similar at extreme amplitudes, and also reported that intense dissipation is likely accompanied by high enstrophy, and that intense enstrophy is not necessarily accompanied by high dissipation for moderate enstrophy, but is for extreme enstrophy. The left and right tails of the PDF of $\tilde{\epsilon }/\nu \tilde{\Omega }$ are well described by the F distribution [20], for which the tails also follow a power law. $P(\tilde{\epsilon }/\bar{\epsilon })$ decreases more rapidly than $P(\tilde{\Omega }/\bar{\Omega })$ for low to moderate amplitudes. When the dissipation and enstrophy are normalized by their extreme values, the tails of both PDFs are well collapsed [26]. However, there appears to be no satisfactory explanation for these observations even in the Gaussian state. The arguments and DNS data on PDF variation from the Gaussian to turbulent state provided in the previous section may lead to a method for answering these questions.

Let us consider the PDF of enstrophy, which is defined by

6.1 $$\tilde{\Omega }=\omega_{ij}^{2}={\left(\frac{\partial u_{i}}{\partial x_{j}}-\frac{\partial u_{j}}{\partial x_{i}}\right)}^{2}.$$

Since $\omega_{ij}$ is an antisymmetric tensor, and d(d−1)/2 terms in d dimensional space contribute to the enstrophy of the Gaussian random field, the enstrophy PDF is the same as that of the passive scalar (5.2) and

6.2 $${\bar{P}}^{G}(\Omega )\;\text{d}\Omega =\frac{{\left(3/2\right)}^{3/2}}{\Gamma (3/2)}\;\Omega^{1/2}\exp\left(-\frac{3}{2}\Omega \right)\;\text{d}\Omega ,\;\Omega =\frac{\tilde{\Omega }}{\bar{\Omega }},$$

in three-dimensional space, where $\bar{\Omega }$ is the mean enstrophy. Indeed, the PDF $\bar{P}(\Omega )$ of run 128A (lowest Reynolds number) follows this theoretical curve as shown in figure 7*d*. The left tail is the power law with the exponent 1/2 irrespective of the Reynolds numbers, again meaning the universality of the left tail, and the right tail decays slightly faster than $\bar{P}(\epsilon_{\theta })$ but slower than $\bar{P}(\epsilon )$ in the present data. Interestingly, the decay factor of the exponential of ${\bar{P}}^{G}(\Omega )$ is 3/2, which is smaller than 5/2 in ${\bar{P}}^{G}(\epsilon )$, where $\epsilon =\epsilon_{u}$ hereafter. Thus, when the Reynolds number is low to moderate, the PDF tails are

6.3 $$\bar{P}(\epsilon )<\bar{P}(\Omega ).$$

Suppose that when the Reynolds number is very large, the PDFs are asymptotically of the following form:

6.4 $$\bar{P}(\epsilon )\;\text{d}\epsilon \propto \epsilon^{3/2}\exp(-C_{\epsilon }\epsilon^{\beta_{\epsilon }})\;\text{d}\epsilon =\epsilon^{3/2}\exp(-\phi (\epsilon ))\;\text{d}\epsilon$$

and

6.5 $$\bar{P}(\Omega )\;\text{d}\Omega \propto \Omega^{1/2}\exp(-C_{\Omega }\Omega^{\beta_{\Omega }})\;\text{d}\Omega =\Omega^{1/2}\exp(-\psi (\Omega ))\;\text{d}\Omega ,$$

where $C_{A}(>0)$ (A=ϵ,Ω). Although these functional forms may differ from those of actual PDFs near their peak region, they are reasonable for small and large amplitudes far from the peak, as shown by the DNS data of previous studies [17,20,26] and the present DNS data. As seen from equations (5.1) and (6.2) and figure 7, when $R_{\lambda }<1$, the exponents $\beta_{A}$ are very close to unity, and begin to slowly decrease with $R_{\lambda }$. By contrast, $C_{A}$ slowly increases from $C_{\epsilon }(0)=5/2$ and $C_{\Omega }(0)=3/2$ in the present study. When $R_{\lambda }$ is very large, the far tails of both PDFs are of the stretched exponential form, with the same exponents $\beta_{\epsilon }=\beta_{\Omega }$ [26]; however, the amplitudes of their PDF plots are normalized by extreme values $\tilde{\epsilon }/\epsilon_{\text{ext}}$ and $\tilde{\Omega }/\Omega_{\text{ext}}$, whereas those in the present study are normalized by the mean $\tilde{\epsilon }/{\bar{\epsilon }}_{u}$ and $\tilde{\Omega }/\bar{\Omega }$. The variation of the decay rates $C_{A}$ with the Reynolds number may or may not be the same. Among many possibilities, it is interesting to consider the following possible scenarios: (1) $C_{\epsilon }$ and $C_{\Omega }$ increase in such a way that the ratio $C_{\epsilon }(R_{\lambda })/C_{\Omega }(R_{\lambda })$ is constant as (5/2)/(3/2)=5/3 or (2) $C_{\epsilon }$ and $C_{\Omega }$ increase differently, but their ratio approaches unity for large amplitudes at large Reynolds numbers.

Scenario (1) reflects the fact that the dissipation and enstrophy comprise five and three components, respectively, which is a firm constraint. When all terms ${\text{e}}_{ij}^{2}$ or $\omega_{ij}^{2}$ behave similarly near the singularity [22,24], they contribute equally to the dissipation and enstrophy, implying that the larger numbers of terms the weaker the fluctuations become via the cancelling of fluctuations that leads to $\bar{P}(\epsilon )<\bar{P}(\Omega )$, with a ratio of about $C_{\epsilon }/C_{\Omega }=5/3$. The number of terms (degrees of freedom in the dissipation and enstrophy) determines the statistics, which is readily understood in the context of the central limit theorem for very large number of terms in the large spatial dimensions, as discussed below. Scenario (2) occurs when the off-diagonal components of ${\text{e}}_{\alpha \beta }^{2}$ (α≠β) dominate the diagonal part ${\text{e}}_{\alpha \alpha }^{2}$ (no summation) near the singularity, and ${\text{e}}_{\alpha \beta }^{2}∼\omega_{\alpha \beta }^{2}$ (for example, in a locally two-dimensional simple but high shear flow $u_{x}=ay$, $e_{12}=e_{21}=a/2$ and $\omega_{12}=-\omega_{21}=a/2$). In this case, three components contribute to dissipation and enstrophy with similar degrees of fluctuation intensity, leading to $\bar{P}(\epsilon )∼\bar{P}(\Omega )$, such that $C_{\epsilon }(R_{\lambda })/C_{\Omega }(R_{\lambda })=1$ describes the far tails of the PDFs at large Reynolds numbers.

**Figure 8.**
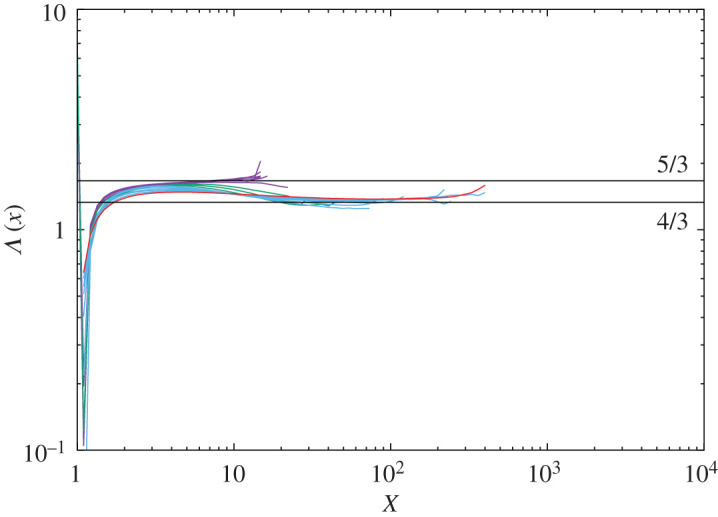
Variation of Λ(x) (equation (6.6)) with increase of the Reynolds number Magenta: 128A-F, green: 256A-C, blue: 512A-F, orange: 1024A. Horizontal lines are 5/3 and 4/3. (Online version in colour.)

In high-Reynolds number turbulence, we infer that both scenarios coexist. For low to moderate dissipation and enstrophy amplitudes, scenario (1) applies and for very large amplitudes, scenario (2) applies. Figure 8 indicates the ratio of the exponential functions defined in equations (6.4) and (6.5)

6.6 $$\Lambda (x)=\frac{\phi_{\epsilon }(x)}{\psi_{\Omega }(x)}.$$

Curves of magenta are for (128A-128F), green for (256A-256C), blue for (512A-512F) and red for 1024A. The curves for low Reynolds numbers (magenta) are close to 5/3, the green curves are in transition state between 5/3 and 4/3, and the curves of blue and red are once close to 5/3 and turn to approach 4/3 (but explanation for 4/3 is not known). Also the tails of the curves tend to be horizontal, which implies that $\beta_{\epsilon }=\beta_{\Omega }$. This behaviour is consistent with those found in the previous studies such as PDF plots in figure 10 and the ratio $b^{\prime }\Omega /b_{\epsilon }^{\prime }$ shown in table IV in [17], and figs 4 and 6 in [20]. Although whether Λ(x) tends to unity for high Reynolds number needs confirmation, the above data and arguments imply that for low to moderate amplitudes, extrapolation from the Gaussian statistics is effective, whereas the Navier–Stokes dynamics generating the singularity determine the far tails of the PDFs.

It is useful and suggestive to consider the effects of the spatial dimension d on the PDFs of the dissipation rates and enstrophy. The number of contributing terms is $N_{\epsilon }=(d-1)(d+2)/2$ for the dissipation rate, $N_{\Omega }=d(d-1)/2$ for the enstrophy, and $N_{\epsilon_{\theta }}=d$ for the passive scalar dissipation rate of the Gaussian random fields, as derived in the appendix. When d=3, $N_{\Omega }=N_{\epsilon_{\theta }}=3$, but when d becomes large, $N_{\epsilon }$ and $N_{\Omega }$ increase in proportion to $d^{2}$, whereas $N_{\epsilon_{\theta }}$ grows linearly in d. This result suggests that ${\bar{P}}_{d=4}(\epsilon )<{\bar{P}}_{d=3}(\epsilon )$ for low to moderate amplitudes of ϵ. Indeed, this prediction is consistent with four-dimensional DNS results [27,28]. Since the exponents of the algebraic pre-factors also increase with d, ${\bar{P}}_{d}(\epsilon )$ and ${\bar{P}}_{d}(\Omega )$ for large d would be characterized by sharp peaks at their means, and would be very narrow at low to moderate Reynolds numbers. The difference $N_{\epsilon }-N_{\Omega }=d-1$ increases with d; thus, ${\bar{P}}_{d}(\epsilon )$ at large amplitudes is much smaller than ${\bar{P}}_{d}(\Omega )$ when compared with the three-dimensional case. By contrast, ${\bar{P}}_{d}(\epsilon_{\theta })$ is much wider than either of these PDFs when $R_{\lambda }$ is not large, due to the smaller decay rate of the exponential function; we anticipate that this trend will hold for high Reynolds numbers.

## 7. Conclusion

In this study, we examined the transition of statistical fluctuations from the Gaussian state to the turbulent state, for gradient fields and dissipation rates of an incompressible velocity, passive vector and passive scalar when $R_{\lambda }$ increases from 0.13 to 110. Kolmogorov scaling of the spectra of the three fields was found to work for $R_{\lambda }>2$. We also found that the transitions of the structure functions of the gradients were not sharp and occurred at about $R_{\lambda }=2∼4$, irrespective of the field type. However, the rate of increase of the structure functions differed, and the structure functions of the passive scalar grew fastest, following a power law, whereas those of the longitudinal velocity gradient increased slowly, with the passive vector being intermediate between them. The dissipation rate moments of the three fields were less scattered than those of the gradients. The transition Reynolds number for the dissipation rate moments was similar to that of the structure functions of the gradients. A contour map of the PDFs revealed that they began to gradually expand at $R_{\lambda }∼4∼10$ for the longitudinal velocity gradient, whereas those of the passive vector and passive scalar showed sharper transitions, expanding at about $R_{\lambda }=4$.

The PDFs of the dissipation rate, and the dissipation rate of the passive scalar, were analytically derived for Gaussian random fluctuations and found to be equivalent to the Gamma distribution, with shape factor α and decay factor β; these are functions of the number of terms in the dissipation rate equations. In three dimensions, (α,β)=(5/2,5/2) for the dissipation rate and (3/2,3/2) for the scalar dissipation rate. When the Reynolds number increased from 0.13 to 110, the left tails of the dissipation rate PDFs consistently obeyed the power law, having the same exponents as those of the Gaussian state; this was constrained by the number of terms, and therefore universal. The PDF peaks shifted towards smaller amplitudes and became higher as the Reynolds number increased. However, the right tails were found to gradually expand.

The enstrophy PDFs for the Gaussian state were also analytically derived as the Gamma distribution with the shape factor (3/2,3/2). We argued that the number of terms included in the dissipation rate and enstrophy equations determines the PDF shape at small amplitudes, which is an important and universal phenomenon that may be used to determine the decay rate of the PDF tails. The PDFs of enstrophy and the scalar dissipation rate, which are computed as the sum of three terms, were wider than that of energy dissipation, which is a five-term sum, at low to moderate Reynolds numbers in the present DNS data. This finding illustrates the usefulness of extrapolation from the Gaussian state to higher Reynolds numbers. Based on the analytical expressions of the dissipation rate and enstrophy PDFs, we conjectured the asymptotic PDFs for large Reynolds number. For low to moderate amplitudes, the enstrophy PDF would decrease more slowly than the dissipation rate PDF, but both PDFs would decay similarly for extreme amplitudes. The effects of the spatial dimensions were discussed and difference among the PDFs of dissipation rate, enstrophy and scalar dissipation rate would become larger as the spatial dimensions, and the scalar dissipation PDF becomes wider, and as the dissipation PDF becomes narrower, with enstrophy being intermediate between them. Actual turbulent fluctuations are far from Gaussian. Further examination and verification of the arguments presented in this study are required.

## Data accessibility

This article has no additional data.

## Appendix A

We consider a multivariate Gaussian random velocity field in d(≥2) dimensional space, which is incompressible, statistically homogeneous and isotropic. The components contributing to the dissipation rate are as follows:

A 1 $$\begin{array}{rl} g_{1} & \;=e_{11},\;g_{2}=e_{22},\;\ldots ,\;g_{d-1}=e_{d-1,d-1}\\ \;\text{and}\;g_{d} & \;=e_{12},\;g_{d+1}=e_{13},\;\ldots ,\;g_{N}=e_{d-1,d},\;N=\frac{1}{2}(d-1)(d+2), \end{array}\}$$

where $e_{ij}$ is the rate of strain tensor in d dimensions and the incompressibility condition is applied. N is the number of terms. The multivariate Gaussian PDF is defined as follows:

A 2 $$P(g)\;\text{d}g=\frac{1}{Z}\exp\left(-\frac{1}{2}\sum_{ij}g_{i}{\left(A^{-1}\right)}_{ij}g_{j}\right)\;\text{d}g,$$

where $Z^{-1}$ is the normalization constant and $A_{ij}$ is the symmetric tensor, with components given by the following correlation $\left\langle g_{i}g_{j}\right\rangle$:

A 3 $$\begin{array}{rl} A & \;=\frac{\bar{\epsilon }/\nu }{d(d+2)}\left[\begin{array}{cc} B & 0\\ 0 & C \end{array}\right] \end{array}$$

A 4 $$\begin{array}{rl} B_{ij} & \;=\delta_{ij}-(1-\delta_{ij})\frac{1}{d-1},\;1\leq i,j\leq d-1 \end{array}$$

A 5 $$\begin{array}{rl} \;\text{and}\;C_{ij} & \;=\frac{d}{2(d-1)},\;d\leq i,j\leq N \end{array}$$

The inverse matrix $A^{-1}$ is given as follows:

A 6 $$\begin{array}{rl} A^{-1} & \;=\frac{2(d-1)(d+2)}{\bar{\epsilon }/\nu }\left[\begin{array}{cc} D & 0\\ 0 & E \end{array}\right], \end{array}$$

A 7 $$\begin{array}{rl} D_{ij} & \;=\delta_{ij}+(1-\delta_{ij})\frac{1}{2},\;1\leq i,j\leq d-1 \end{array}$$

A 8 $$\begin{array}{rl} \;\text{and}\;E_{ij} & \;=\delta_{ij}\;d\leq i,j\leq N. \end{array}$$

Interestingly,

A 9 $$\frac{\epsilon }{\nu }=2{\text{e}}_{ij}^{2}=4\left(\sum_{j=1}^{N}g_{i}^{2}+\sum_{i\neq j=1}^{d-1}g_{i}g_{j}\right)=4gA^{-1}g.$$

Therefore, equation (A 2) is written in terms of the rate of strain tensor normalized by the mean dissipation rate $\zeta ={\left(\bar{\epsilon }/\nu \right)}^{-1/2}g$ as

A 10 $$\bar{P}(\zeta )\;\text{d}\zeta =\frac{1}{Z}\exp\left(-\frac{N}{2}\epsilon \right)\;\text{d}\zeta ,\;\epsilon =\frac{\tilde{\epsilon }}{\bar{\epsilon }}.$$

Since $\bar{P}(\zeta )$ is the joint PDF for N variables, a PDF of ϵ can be obtained by integrating over N−1 variables. To achieve this, we introduce a hyperspherical coordinate as follows:

A 11 $$\begin{array}{rl} \zeta_{1} & \;=\rho \cos\phi_{1},\;\zeta_{2}=\rho \sin\phi_{1}\cos\phi_{2},\;\zeta_{3}=\rho \sin\phi_{1}\sin\phi_{2}\cos\phi_{3},\\ & \;\;\cdots \\ \;\text{and}\;\zeta_{N-1} & \;=\rho \sin\phi_{1}\sin\phi_{2}\cos\phi_{3}\cdots \cos\phi_{N-1},\;\zeta_{N}=\rho \sin\phi_{1}\sin\phi_{2}\cos\phi_{3}\cdots \sin\phi_{N-1}, \end{array}\}$$

where the range of angle ϕ is $0\leq \phi_{i}<\pi$ for i=1,…,N−2 and $0\leq \phi_{N-1}<2\pi$. Substituting equation (A 11) into equation (A 9) and arranging the terms, we obtain

A 12 $$\epsilon =\rho^{2}(1+f(\phi_{1},\phi_{2},\ldots ,\phi_{d-1})),$$

where f is the positive definite function, which is the sum of the products of d−1 trigonometric functions. A further variable change from ρ to ϵ yields

A 13 $$\begin{array}{rl} \bar{P}(\zeta )\;\text{d}\zeta & \;=\bar{P}(\rho ,\phi_{1},\ldots ,\phi_{N-1})\rho^{N-1}\;\text{d}\rho \;\text{d}\Omega_{N-1},\\ & \;=\frac{\epsilon^{\left(N-2\right)/2}}{2\sqrt{1+f(\phi_{1},\ldots ,\phi_{d-1})}}\;\bar{P}(\rho ,\phi_{1},\ldots ,\phi_{N-1})\;\text{d}\epsilon \;\text{d}\Omega_{N-1} \end{array}$$

and

A 14 $$\;\text{d}\Omega_{N-1}=\sin^{N-3}\phi_{1}\sin^{N-4}\phi_{2}\cdots \sin\phi_{N-2}\dot{\;}\text{d}\phi_{1}\cdots \;\text{d}\phi_{N-2}\;\text{d}\phi_{N-1},$$

where $\;\text{d}\Omega_{N-1}$ is the solid angle element of the hypersphere in N−1 dimensions. Substituting equation (A 10) into equation (A 13) and noting that the integral over the solid angles is absorbed into the normalization constant, we finally arrive at

A 15 $$\bar{P}(\epsilon )\;\text{d}\epsilon =\frac{1}{Z_{\epsilon }}\epsilon^{\left(N_{\epsilon }-2\right)/2}\exp\left(-\frac{N_{\epsilon }}{2}\epsilon \right)\;\text{d}\epsilon ,\;N_{\epsilon }=\frac{\left(d-1)(d+2\right)}{2}.$$

This is the Gamma distribution. When d=3, N=5; thus, we obtain equation (5.1). For the passive scalar and enstrophy dissipations, the correlation matrix A is diagonal and the same analysis is applied, such that it is straightforward to obtain

A 16 $$\bar{P}(\Omega )\;\text{d}\Omega =\frac{1}{Z_{\Omega }}\Omega^{\left(N_{\Omega }-2\right)/2}\exp\left(-\frac{N_{\Omega }}{2}\Omega \right)\;\text{d}\Omega ,\;N_{\Omega }=\frac{d(d-1)}{2}$$

and

A 17 $$\bar{P}(\epsilon_{\theta })\;\text{d}\epsilon_{\theta }=\frac{1}{Z_{\epsilon_{\theta }}}\epsilon_{\theta }^{\left(N_{\Omega }-2\right)/2}\exp\left(-\frac{N_{\theta }}{2}\epsilon_{\theta }\right)\;\text{d}\epsilon_{\theta },\;N_{\epsilon_{\theta }}=d.$$
